# An Inventory of Nutrient-Responsive Genes in *Arabidopsis* Root Hairs

**DOI:** 10.3389/fpls.2016.00237

**Published:** 2016-03-01

**Authors:** Jorge E. Salazar-Henao, Wolfgang Schmidt

**Affiliations:** ^1^Institute of Plant and Microbial Biology, Academia SinicaTaipei, Taiwan; ^2^Biotechnology Center, National Chung-Hsing UniversityTaichung, Taiwan; ^3^Genome and Systems Biology Degree Program, College of Life Science, National Taiwan UniversityTaipei, Taiwan

**Keywords:** iron deficiency, phosphate deficiency, root hairs, systems biology, gene regulation

## Abstract

Root hairs, single cell extensions of root epidermal cells that are critically involved in the acquisition of mineral nutrients, have proven to be an excellent model system for studying plant cell growth. More recently, omics-based systems biology approaches have extended the model function of root hairs toward functional genomic studies. While such studies are extremely useful to decipher the complex mechanisms underlying root hair morphogenesis, their importance for the performance and fitness of the plant puts root hairs in the spotlight of research aimed at elucidating aspects with more practical implications. Here, we mined transcriptomic and proteomic surveys to catalog genes that are preferentially expressed in root hairs and responsive to nutritional signals. We refer to this group of genes as the root hair trophomorphome. Our analysis shows that the activity of genes within the trophomorphome is regulated at both the transcriptional and post-transcriptional level with the mode of regulation being related to the function of the gene product. A core set of proteins functioning in cell wall modification and protein transport was defined as the backbone of the trophomorphome. In addition, our study shows that homeostasis of reactive oxygen species and redox regulation plays a key role in root hair trophomorphogenesis.

## Introduction

Root hairs are the major site for the acquisition of mineral nutrients from the soil. In particular, this holds true for nutrients that occur in complexes with soil constituents or in insoluble forms with restricted mass flow to roots, such phosphate (Pi) or ferric iron (Schmidt, 1999; Chiou and Lin, 2011). Not only the increase in absorptive surface area but also the preferential expression of enzymes involved in the mobilization and uptake of essential elements contribute to the role of root hairs as a major conduit for mineral nutrients (Lan et al., 2013; Tanaka et al., 2014).

In *Arabidopsis*, root hairs develop from specialized epidermal cells (trichoblasts) that are located over the anticlinal walls of two underlying cortical cells. The positional-biased cell fate determination leads to longitudinally oriented files of root hair-bearing cells that are interspersed with non-hair cell files (Dolan et al., 1994; Cederholm et al., 2012; Petricka et al., 2012a). The positional signal is transduced by the leucine-rich repeat receptor-like kinase SCRAMBLED (SCM) (Kwak et al., 2005; Kwak and Schiefelbein, 2007, 2008; Hassan et al., 2010). Signal strength, which is influenced by the arrangement of epidermal cells and several feedback loops that increases SCM abundance in trichoblasts, tips the balance of the distribution of two WD-repeat/bHLH/Myb transcriptional complexes that act either as inhibitor or as activator of the root hair cell fate (Kwak and Schiefelbein, 2008, 2014). Subtle changes in this balance leads to accumulation of the homeodomain-leucine-zipper transcription factor GLABRA 2 (GL2) in non-hair cells (Schiefelbein et al., 2009, 2014; Tominaga-Wada et al., 2011; Grebe, 2012). GL2 directly represses a suite of transcriptional regulators that are critical for root hair morphogenesis including the bHLH VIIIc subfamily transcription factor *ROOT HAIR DEFECTIVE 6* (*RHD6*) which is expressed in hair cells (Masucci and Schiefelbein, 1994; Menand et al., 2007; Lin et al., 2015). Application of auxin and ethylene rescues the root hairless phenotype of the *rhd6* mutant, indicative of an alternative pathway to alter cell fate downstream of the WD-repeat/bHLH/Myb transcriptional complexes, a pathway that is likely to be activated by external cues (Masucci and Schiefelbein, 1996).

Tip growth requires a complex machinery of proteins that control actin cytoskeleton dynamics, mediate the synthesis of new cell wall material and the formation and targeting of secretory vesicles to the growing tips (Rounds and Bezanilla, 2013). Omics-based approaches have generated an inventory of transcripts and proteins that preferentially accumulate in root hair cells and have set the stage for a systems-oriented understanding of root hair biology. The first genome-wide transcriptomic approach aimed at dissecting root hair-specific expression pattern reported a comparison of the transcriptome of the root hairless mutant *rhd2* with that of the wild type, yielding a suite of 606 genes that are differentially expressed between the two genotypes (Jones et al., 2006). A series of studies based on reporter-driven labeling of specific cell types and subsequent isolation by fluorescence-activated cell sorting (FACS) of root protoplasts, has produced a detailed spatiotemporal expression atlas of cell type-specific cell identity programs in *Arabidopsis* roots including root hairs (Birnbaum et al., 2003; Brady et al., 2007).

A set of 208 “root-hair core” genes was deduced from comprehensive transcriptional profiling of epidermal cells from several cell-fate mutants and hormone-treated plants and organized into a gene regulatory network of root epidermis cell differentiation (Bruex et al., 2012). A FACS-based RNA-seq analysis of *Arabidopsis* root hairs identified 20,822 expressed genes in root hairs; transcripts of 1,617 genes accumulated differentially between root hairs and non-root hair tissues (all root tissues except root hairs). About 4% of the transcripts showed a root hair-specific expression and were not detected in other tissues (Lan et al., 2013).

A comparison of the transcriptomes of pollen and root hairs revealed a common set of genes that define an “apical growth core,” comprised of 277 genes that play critical roles in the extension of tip-growing cells. Genes in this set encode proteins that are mainly involved in responses to reactive oxygen species, small GTPase signaling, vesicle-mediated transport and biopolymer modification (Becker et al., 2014).

Although improved mass spectrometry methodologies have dramatically increased the resolution of proteomic profiling, the number of detected proteins in a given cell type or tissue is still substantially lower than that of the identified transcripts. In *Arabidopsis*, high-resolution proteome analysis led to the identification of about 300 proteins that accumulated differentially between root hairs and other cell types (Petricka et al., 2012b; Lan et al., 2013).

A comparison of mRNA and protein profiles of root hairs revealed a relatively low concordance between the two layers, indicative of substantial regulatory intervention at the protein level (Lan et al., 2013). This discrepancy may partially result from extensive, cell type-specific alternative splicing that tunes the amount of translated transcripts (Lan et al., 2013). Intron retention often leads to the inclusion of premature termination codons and subsequence degradation of the mRNA via the nonsense-mediated decay RNA surveillance pathway (Drechsel et al., 2013). In support of this supposition, transcripts derived from genes that are tightly co-expressed in root hairs and likely to be critically involved in root hair morphogenesis showed less pronounced intron retention (i.e., higher splicing fidelity) than genes that were not co-regulated (Lan et al., 2013). A regulatory role of alternative splicing in root hair morphogenesis is further supported by the preferential expression of several splicing factors, proteins with mRNA-binding domains putatively involved in alternative splicing, and proteins involved in other RNA-related processes such as regulation of mRNA stability in soybean root hairs (Brechenmacher et al., 2012).

While the program that controls root hair cell fate and morphogenesis is genetically fixed, the shape, length and density of hairs is strongly affected by the prevailing environmental conditions. In particular, mineral nutrients with low mobility such as Pi, Fe, and Mn can alter the root hair phenotype (Ma et al., 2001; Müller and Schmidt, 2004; Yang et al., 2008). We assume that the proteins executing intrinsic developmental programs are largely functionally congruent with those that are recruited to induce the phenotype that is typical of a given nutrient regime. However, some proteins with seemingly redundant functions might be more responsive to environmental signals than others. For example, the paralogous R3 MYB proteins CAPRICE (CPC), ENHANCER OF TRY AND CPC1 (ETC1), and TRYPTICHON (TRY) that act redundantly as positive regulators of the root hair cell fate as part of a WD-repeat/bHLH/Myb complex (Kirik et al., 2004; Schellmann et al., 2007; Simon et al., 2007), acquire additional functions when plant were subjected to Pi deficiency (Chen and Schmidt, 2015). Thus, the concept of genetic redundancy might not apply to all nutritional contexts. In the present investigation, we mined public data sets that comprise transcriptomic and proteomic data on expression of root hair genes and genes that are responsive to either Pi or Fe deficiency. This survey revealed a set of nutrient-responsive root hair genes, here designated as the “trophomorphome” that are critically involved in altering root hair morphogenesis to tune developmental programs to the prevailing conditions.

## Results and discussion

### Nutrient deficiency-induced changes in the root hair transcriptome

**Figure 1:**
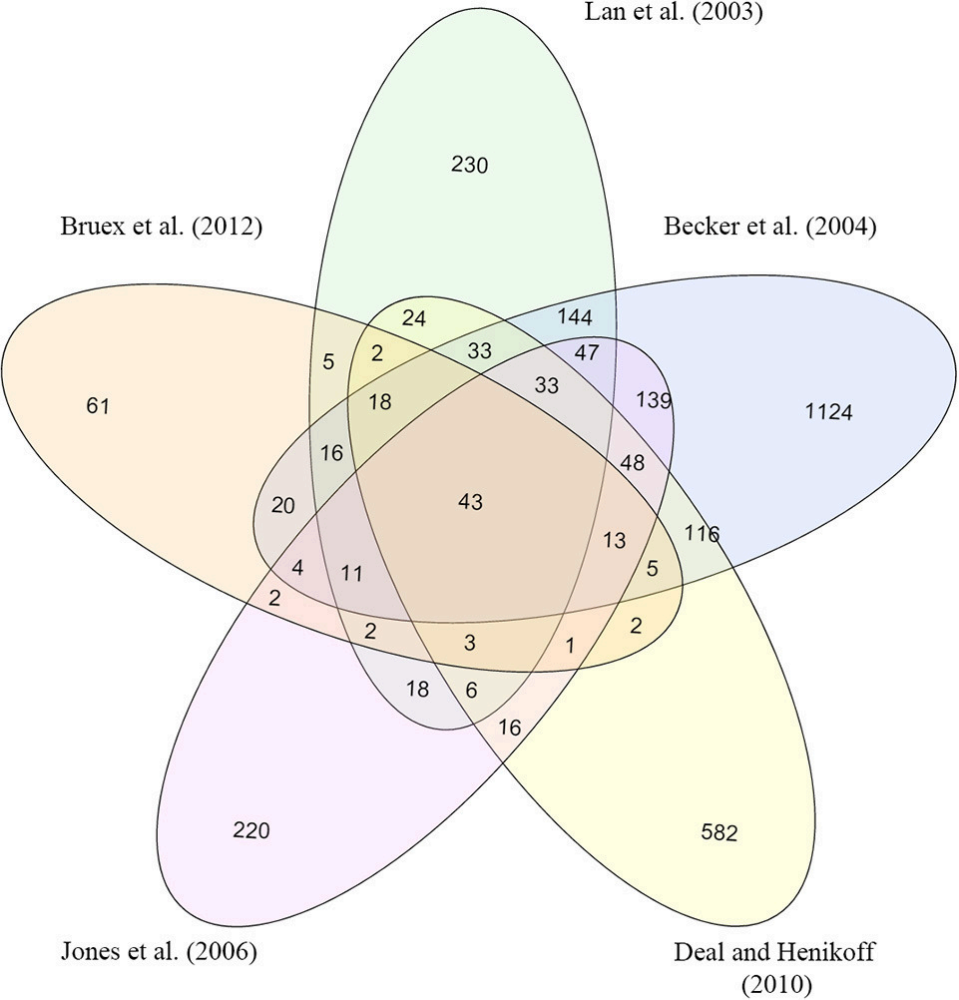
**Venn diagram showing the overlap of genes that are preferentially expressed in *Arabidopsis* root hairs as identified in five studies (Jones et al., 2006; Deal and Henikoff, 2010; Bruex et al., 2012; Lan et al., 2013; Becker et al., 2014)**.

Transcripts that were robustly detectable in root hairs were considered as a basis to identify genes that are involved in root hair trophomorphogenesis. Here, genes were defined as being robustly detectable if they were reported in at least four of five studies that catalog transcripts of genes that are preferentially expressed in root hairs (Jones et al., 2006; Deal and Henikoff, 2010; Bruex et al., 2012; Lan et al., 2013; Becker et al., 2014). In total, 107 genes fulfilled this criterion (Figure 1). From this group, 37 genes were reported to be responsive to Pi starvation at the transcript and/or protein level, five genes were responsive to Fe deficiency, and the expression of three genes was affected in both growth types (Table 1; Lan et al., 2012a; Rodríguez-Celma et al., 2013; Pan et al., 2015). For most of the genes, growth type-dependent regulation occurred primarily at the transcript level; only 10 genes were increased in abundance as transcripts and as proteins upon nutrient starvation. Notably, for two genes, *RSH19* and *FLA6*, only the protein but not the transcript level changed when plants were grown on Pi-deficient media, indicative of chiefly post-transcriptional gene regulation (Table 1).

**Table 1:** **Pi- and Fe-responsive genes that are preferentially expressed in root hairs**.

**Locus**	**Gene name**	**-Pi**	**Aux**	**RSL4**	**-Fe**
At3g60330	AHA7, HA7, H(+)-ATPase 7				
At1g34760	RHS5, ROOT HAIR SPECIFIC 5				
At4g19680	IRON REGULATED TRANSPORTER 2, IRT2				
**At4g02270**	RHS13, ROOT HAIR SPECIFIC 13				
**At1g30870**	Peroxidase superfamily protein				
At5g61550	U-box domain-containing protein kinase family protein				
At4g25820	XTH14, XYLOGLUCAN ENDOTRANSGLUCOSYLASE/HYDROLASE 14				
At1g48930	GH9C1, GLYCOSYL HYDROLASE 9C1				
At5g05500	PROLINE-RICH PROTEIN-LIKE 1, PRPL1				
**At4g26010**	Peroxidase superfamily protein				
At1g12560	EXP7, EXPANSIN A7				
At4g13390	EXT12, EXTENSIN 12				
At1g62980	EXP18, EXPANSIN 18				
At4g40090	AGP3, ARABINOGALACTAN PROTEIN 3				
At5g57530	XTH12, XYLOGLUCAN ENDOTRANSGLUCOSYLASE/HYDROLASE 12				
At4g28850	XTH26, XYLOGLUCAN ENDOTRANSGLUCOSYLASE/HYDROLASE 26				
At1g30850	ROOT HAIR SPECIFIC 4, RSH4				
At3g10710	RHS12, ROOT HAIR SPECIFIC 12				
**At2g37670**	Transducin/WD40 repeat-like superfamily protein				
At5g40860	Unknown protein				
At3g12540	Protein of unknown function, DUF547				
At4g34580	CAN OF WORMS1, COW1				
At5g65090	DEFORMED ROOT HAIRS 4, DER4				
At2g41970	Protein kinase superfamily protein				
At1g12950	ROOT HAIR SPECIFIC 2, RSH2				
At4g24580	REN1, ROP1 ENHANCER 1				
At4g27290	S-locus lectin protein kinase family protein				
At5g58010	LJRHL1-LIKE 3, LRL3				
At2g46860	PPA3, PYROPHOSPHORYLASE 3				
At4g22080	RHS14, ROOT HAIR SPECIFIC 14				
**At3g23190**	HR-like lesion-inducing protein-related				
At4g31250	Leucine-rich repeat protein kinase family protein				
At5g01280	BASIC PROLINE-RICH PROTEIN3, BPP3				
At4g16350	CALCINEURIN B-LIKE PROTEIN 6, CBL6				
At4g25160	PUB35				
At4g30670	Putative membrane lipoprotein				
At5g40510	Sucrase/ferredoxin-like family protein				
At5g67400	RHS19, ROOT HAIR SPECIFIC 19	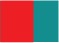			
At2g20520	FASCICLIN-LIKE ARABINOGALACTAN 6, FLA6	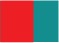			

Locus identifiers shown in bold letters indicate genes for which both mRNA and protein accumulate in root hairs. Dark green labels denote genes that are up-regulated in response to nutrient starvation at both the mRNA and protein level; light green labels indicate genes that are up-regulated only at the transcript level. Red/dark green labels indicate genes that are not responsive to nutrient starvation at the mRNA level but are up-regulated at the protein level. Aux, auxin-responsive.

**Figure 2:**
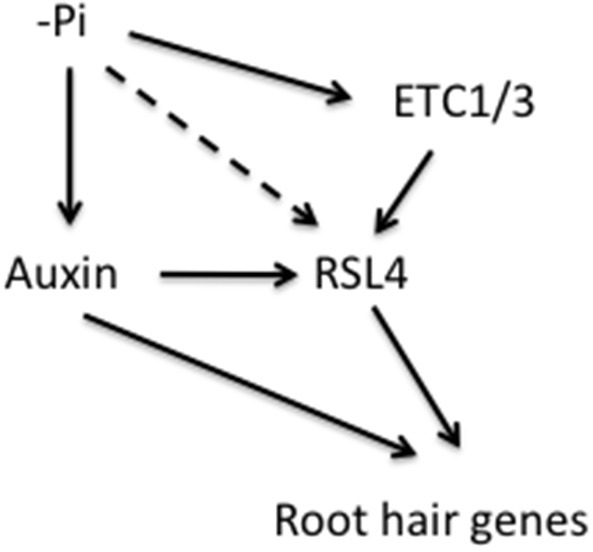
**Possible pathways for the effect of phosphate (Pi) availability on root hair trophomorhogenesis**. Pi starvation increases the responsiveness to auxin, which in turn positively regulate the bHLH transcription factor RSL4. The R3 MYB proteins ETC1 and ETC3, which are both up-regulated by Pi starvation affect RSL4 abundance. In addition, RSL4 could be regulated by Pi starvation via an auxin- and ETC-independent pathway. RSL4 directly regulates a suite of root hair morphogenesis genes. Some auxin-regulated root hair genes do not require functional RSL4. Straight lines indicate experimentally verified reactions and dotted lines denote hypothetical pathways.

Environmental information can affect root hair morphogenesis at different stages of the developmental pathway (Figure 2). The best-explored example for environmentally induced changes in root hair morphogenesis is the response to insufficient Pi availability. Pi starvation leads to the induction of the *CPC* paralogs *ETC1* and *ETC3* that negatively regulate *GL2* expression and thus support the root hair cell fate (Schiefelbein et al., 2009; Lan et al., 2012a; Savage et al., 2013; Tominaga-Wada and Wada, 2014). Expression of *ETC1* is also affected by modifying the activity of HISTONE DEACETYLASE 6 (HDA6), which is associated with altered epidermal patterning (Li et al., 2015). *HDA6* is responsive to various hormones and abiotic factors (Luo et al., 2012; Liu et al., 2014), indicating that environmental signals can alter cell fate assignment *via* histone modifications and subsequently altered expression of cell specification genes such as *ETC1*.

The plant hormone auxin is another important player in the integration of environmental information into the morphogenetic pathway of root hairs. Auxin is though to impact cell fate and differentiation downstream of RHD6 (Masucci and Schiefelbein, 1996). Recently, auxin-inducible genes involved in root hair morphogenesis were identified by subjecting *rdh6* plants to external auxin and subsequent transcriptomic analysis (Bruex et al., 2012). Notably, all of the auxin-inducible genes were preferentially expressed in root hairs and the expression of many of these genes was dependent on functional RHD6. This led to the assumption that RHD6 positively regulates the sensitivity to auxin (Bruex et al., 2012). Twenty-six of the robustly expressed root hair genes are auxin inducible (Table 1; Bruex et al., 2012). All of the auxin-responsive genes are responsive to Pi deficiency, but not all Pi-responsive genes are responsive to Pi, indicating that Pi acts upstream of auxin (Table 1). In support of this assumption, it has been shown that Pi deficiency increases auxin-responsiveness (Lan et al., 2012b).

The bHLH transcription factor ROOT HAIR DEFECTIVE 6-LIKE 4 (RSL4) is expressed in hair cells prior to initiation of root hair outgrowth and regulates a suite of genes encoding hydrolytic enzymes that aid in modifying cell walls to allow rapid tip growth of the cell (Menand et al., 2007; Yi et al., 2010; Datta et al., 2015).

RSL4 is a direct target of RHD6 and responsive to Pi starvation both at the transcriptional and post-translational level; the half-life of RSL4 protein is significantly increased when plants were grown on Pi-deplete media (Yi et al., 2010; Datta et al., 2015). A positive correlation between the abundance of RSL4 and root hair length was also observed in wheat (Han et al., 2016), indicating that RSL4 orthologs are key factors in root hair morphogenesis. In the present survey, 21 genes were reportedly dependent on RSL4 (Table 1). From this subset, all but four genes are responsive to auxin, suggesting that RSL4 is exerting its action mainly via the auxin pathway. On the other hand, nine auxin-inducible genes are RSL4-independent, indicative of multiple auxin pathways that can affect the expression of root hair genes (Figure 2; Table 1). The relative small group of Fe-responsive genes does not appear to affect root hair development chiefly via auxin; only *HA7* is within the group of auxin-inducible genes (Table 1).

### Nutrient deficiency-induced changes in the root hair proteome

Two reference maps of proteins that are preferentially or exclusively expressed in *Arabidopsis* root hairs are available, comprising 238 and 71 proteins (Petricka et al., 2012b; Lan et al., 2013). Since the overlap between the two surveys is very small (seven proteins), the two sets were merged for the current analysis. The so defined root hair proteome contains 309 proteins, of which 35 were responsive to Pi starvation and 22 to Fe deficiency (Table 2). Induction by auxin was reported for nine of the proteins, two proteins are dependent on functional RSL4. Notably, all of the seven proteins that were detected in both data sets were responsive to Pi starvation. Also of note, only for five proteins a cognate root hair-specific transcript was detected. Thus, under control (Pi- and Fe-replete) conditions, most of the root hair proteins are post-transcriptionally regulated.

**Table 2:** **Gene loci of Pi- and Fe-responsive proteins that are preferentially expressed in root hairs**.

**Locus**	**Gene name**	**−Pi**	**−Fe**	**Aux**	**RSL4**
**At4g02270^L^**	RHS13, ROOT HAIR SPECIFIC 13				
**At1g30870^L,P^**	Peroxidase superfamily protein				
At1g56550^L,P^	RGXT3, RHAMNOGALACTURONAN SPECIFIC XYLOSYLTRANSFERASE 3				
At4g09990^L,P^	LUCURONOXYLAN METHYLTRANSFERASE 2, GXM2				
At5g49270^L,P^	COBL9, COBRA-LIKE 9				
At2g47540^L,P^	Pollen Ole e 1 allergen and extensin family protein				
At3g01190^L,P^	Peroxidase superfamily protein				
At2g27190^P^	PAP12, PURPLE ACID PHOSPHATASE 12				
At1g05240^L^	Peroxidase superfamily protein PPP				
At5g01220^P^	SQD2, SULFOQUINOVOSYLDIACYLGLYCEROL 2				
At3g01290^P^	HIR2, HYPERSENSITIVE INDUCED REACTION 2		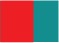		
At2g34585^P^	Unknown protein				
At3g52190^P^	PHF1, PHOSPHATE TRANSPORTER TRAFFIC FACILITATOR 1				
At4g00100^P^	RIBOSOMAL PROTEIN S13A, RPS13				
At5g17820^L^	Peroxidase superfamily protein				
At3g48890^P^	MAPR3, MEMBRANE STEROID BINDING PROTEIN 2				
**At5g04960^P^**	Plant invertase/pectin methylesterase inhibitor superfamily				
**At2g37670^P^**	Transducin/WD40 repeat-like superfamily protein				
At5g18900^P^	2-oxoglutarate (2OG) and Fe(II)-dependent oxygenase superfamily protein				
**At4g26010^P^**	Peroxidase superfamily protein				
At1g49140^P^	Complex I subunit NDUFS6				
At3g57300^L^	INO80, INO80 ORTHOLOG				
At3g63190^P^	RIBOSOME RECYCLING FACTOR				
At1g04810^P^	26S proteasome regulatory complex				
At4g34180^P^	CYCLASE1				
At1g76690^P^	12-OXOPHYTODIENOATE REDUCTASE 2, OPR2				
**At3g23190^P^**	HR-like lesion-inducing protein-related				
At3g19390^L^	Granulin repeat cysteine protease family protein				
At2g45070^P^	SEC61 BETA, SUPPRESSORS OF SECRETION-DEFECTIVE 61 BETA	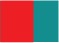	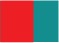		
At3g17910^P^	EMBRYO DEFECTIVE 3121, SURF1, SURFEIT 1	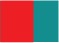			
At3g57630^P^	Exostosin family protein;	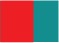			
At5g14060^P^	CARAB-AK-LYS	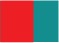			
At5g46160^P^	Ribosomal protein L14p/L23e family protein	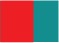			
At1g15710^P^	Prephenate dehydrogenase family protein	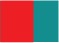			
At5g59910^P^	Histone H2B, HTB4	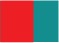			
At3g08550^P^	ELD1, ELONGATION DEFECTIVE 1	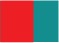			
At3g23600^P^	Alpha/beta-hydrolases superfamily protein				
At4g11600^P^	GLUTATHIONE PEROXIDASE 6, GPX6				
At3g27890^P^	NADPH:QUINONE OXIDOREDUCTASE, NQR				
At1g29250^P^	Alba DNA/RNA-binding protein				
At5g56350^P^	Pyruvate kinase family protein				
At1g13930^L^	Involved in response to salt stress		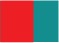		
At1g08450^P^	CALRETICULIN 3, CRT3		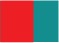		
At5g23740^P^	RIBOSOMAL PROTEIN S11-BETA, RPS11-BETA		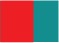		
At3g29250^P^	SDR4, SHORT-CHAIN DEHYDROGENASE REDUCTASE 4		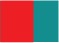		
At1g54410^L^	DEHYDRIN 11KDA, HIRD11		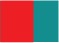		
At1g70410^P^	BCA4, BETA CARBONIC ANHYDRASE 4		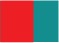		

Locus identifiers shown in bold letters indicate genes for which both mRNA and protein accumulate in root hairs. Dark green labels denote genes that are up-regulated in response to nutrient starvation at both the mRNA and protein level; light green labels indicate genes that are up-regulated only at the transcript level. Red/dark green labels indicate genes that are not responsive to nutrient starvation at the mRNA level, but are up-regulated at the protein level. ^L^, described in Lan et al. (2013); ^P^, described in Petricka et al. (2012b); Aux, auxin-responsive.

Interestingly, none of the 56 proteins that are specifically expressed in epidermal non-hair cells (Petricka et al., 2012b) are responsive to either Pi- or Fe-deficient conditions.

### Genes that accumulate in root hairs at the mRNA and protein level are disparately regulated

**Figure 3:**
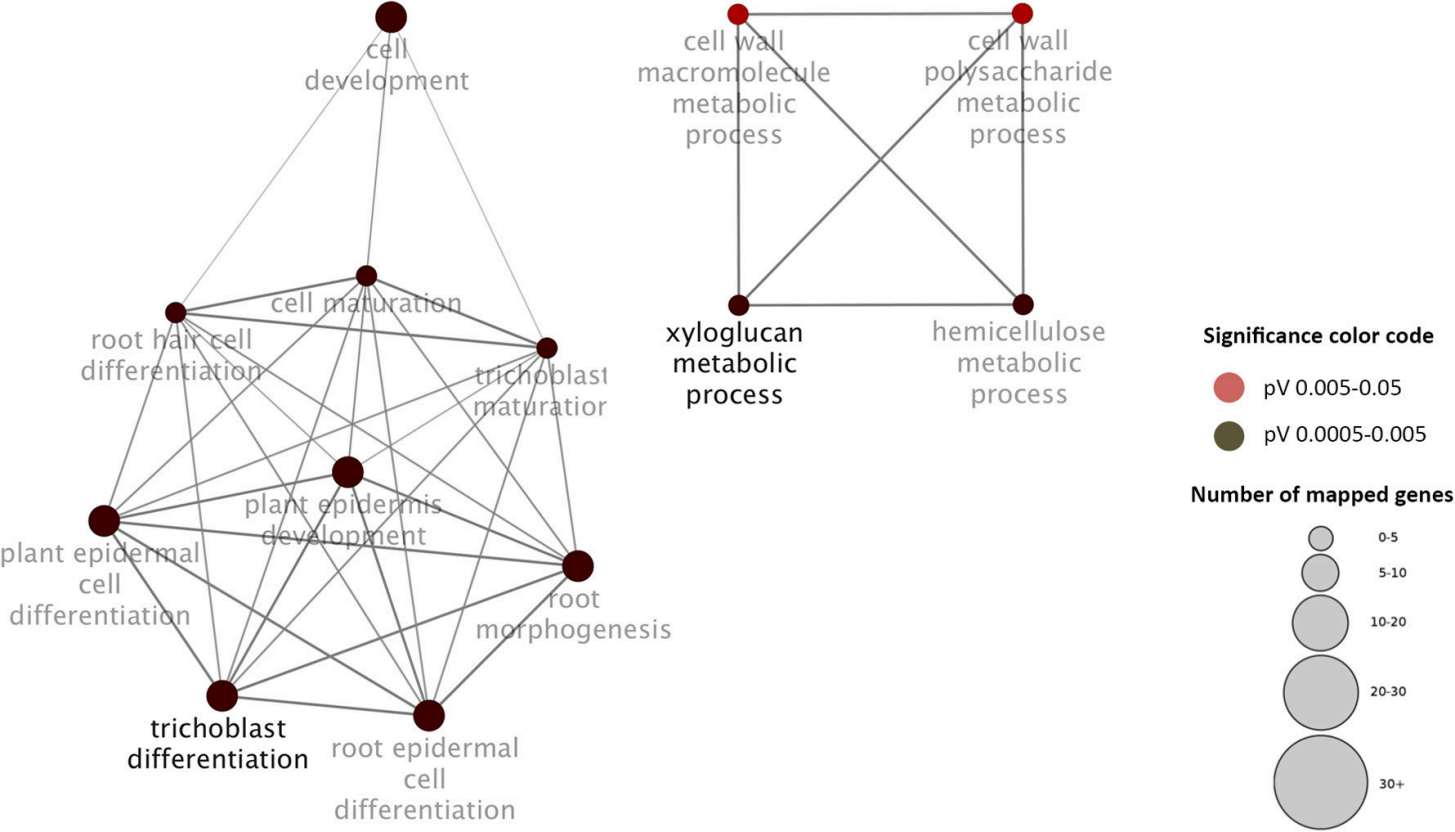
**Visualization the non-redundant biological gene ontology terms for genes that accumulate in root hairs at the transcript level**. The size of the nodes corresponds to the number of the genes associated with a term. The significance is represented by the color of the nodes. Networks were constructed by ClueGo and displayed in “significance view” by Cytoscape (http://apps.cytoscape.org/apps/cluego).

**Figure 4:**
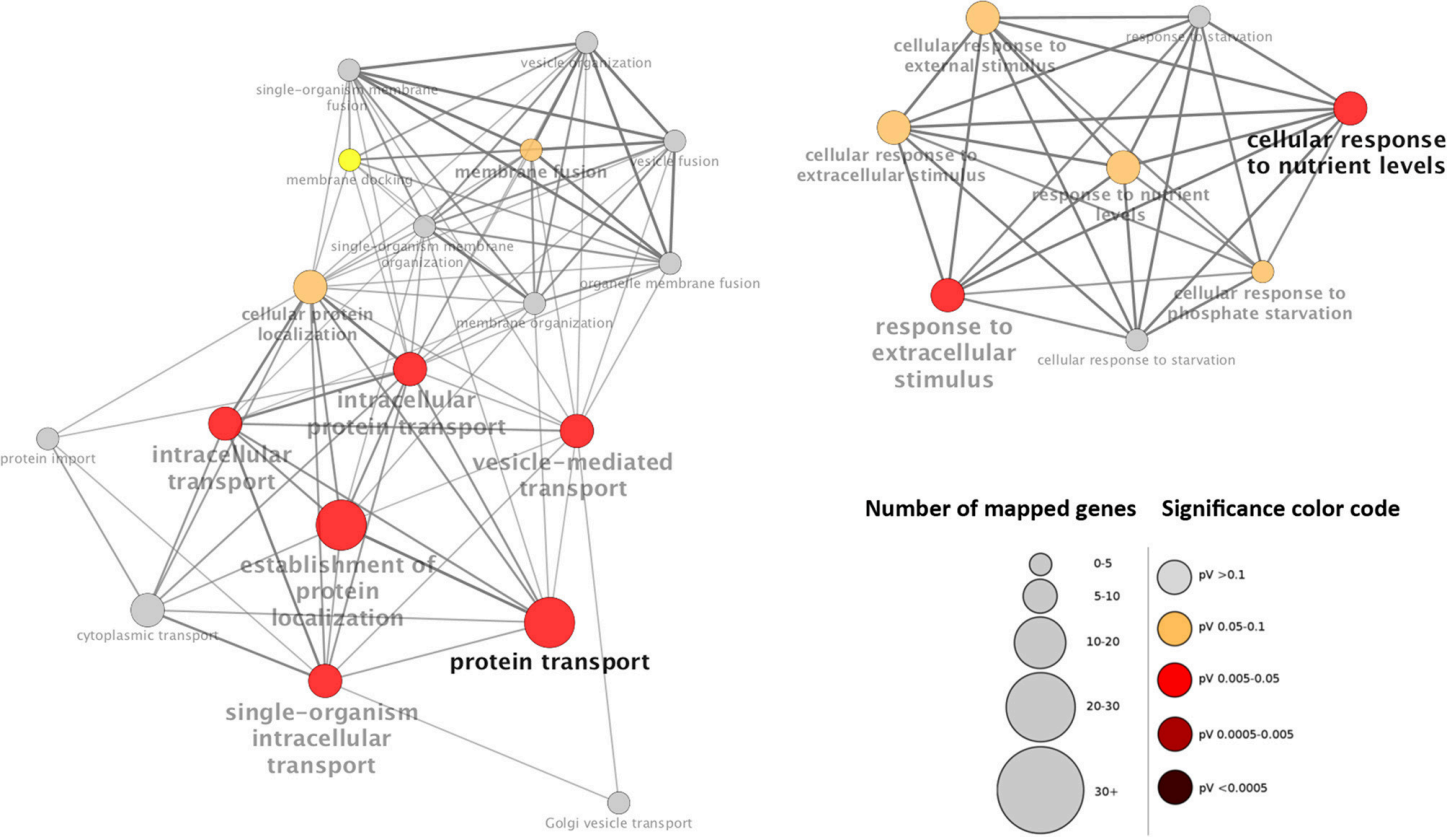
**Visualization the non-redundant biological gene ontology terms for proteins that accumulate in root hairs**. The size of the nodes corresponds to the number of the genes associated to a term. The significance is represented by the color of the nodes. Networks were constructed by ClueGo and displayed in “significance view” by Cytoscape (http://apps.cytoscape.org/apps/cluego).

Contrary to expectations, the main functions differed markedly between genes that accumulated in root hairs either at the transcript or protein level. While for mRNAs the gene ontology categories “trichoblast differentiation” and related processes are highly overrepresented, root hair proteins are functionally related to “protein transport” and “cellular response to nutrient levels” (Figures 3, 4).

The functional incongruity between the two levels is surprising and can be caused by several factors. Generally, mRNA and protein levels are only moderately correlated (Vogel and Marcotte, 2012). For the present case, several non-mutually exclusive scenarios can be considered. Firstly, pronounced transcript changes might be translated into relatively subtle changes in protein levels that are below the threshold for being classified as differentially expressed but that may still be important for root hair morphogenesis. Secondly, gene activity may be mainly regulated via protein turnover, while the steady-state abundance of the cognate transcripts remains stable. Another possibility is that transcription and translation occur at different developmental stages, resulting in a temporal decoupling of mRNA and protein levels. Also, translation in root hair cells might differ in efficiency relative to other tissues, causing proteins to accumulate in root hairs without their cognate transcripts (i.e. transcripts are not significantly more or less abundant in hair cells over other tissues). Finally, the detection of proteins in root hairs without corresponding transcripts might partly be due to proteins migrating into trichoblasts from neighboring cells.

A tempting assumption is that the disparate regulation of root hair genes is related to their function. Genes with root hair-specific accumulation of mRNAs are largely related to polysaccharide metabolism and trichoblast development. In this group, transcripts appear to be stable and genes might be mainly regulated at the transcriptional level. In contrast, genes that mainly function in protein transport, membrane fusion and responses to nutrient level are chiefly protein-level regulated. This supposition is supported by the fact that in the protein group several genes are regulated at the protein level in response to nutritional signals, while no transcript changes were observed in response to nutrient starvation. In the group of chiefly transcriptionally regulated genes, such a pattern was only observed for two genes (Tables 1, 2).

### The core trophomorphome

**Figure 5:**
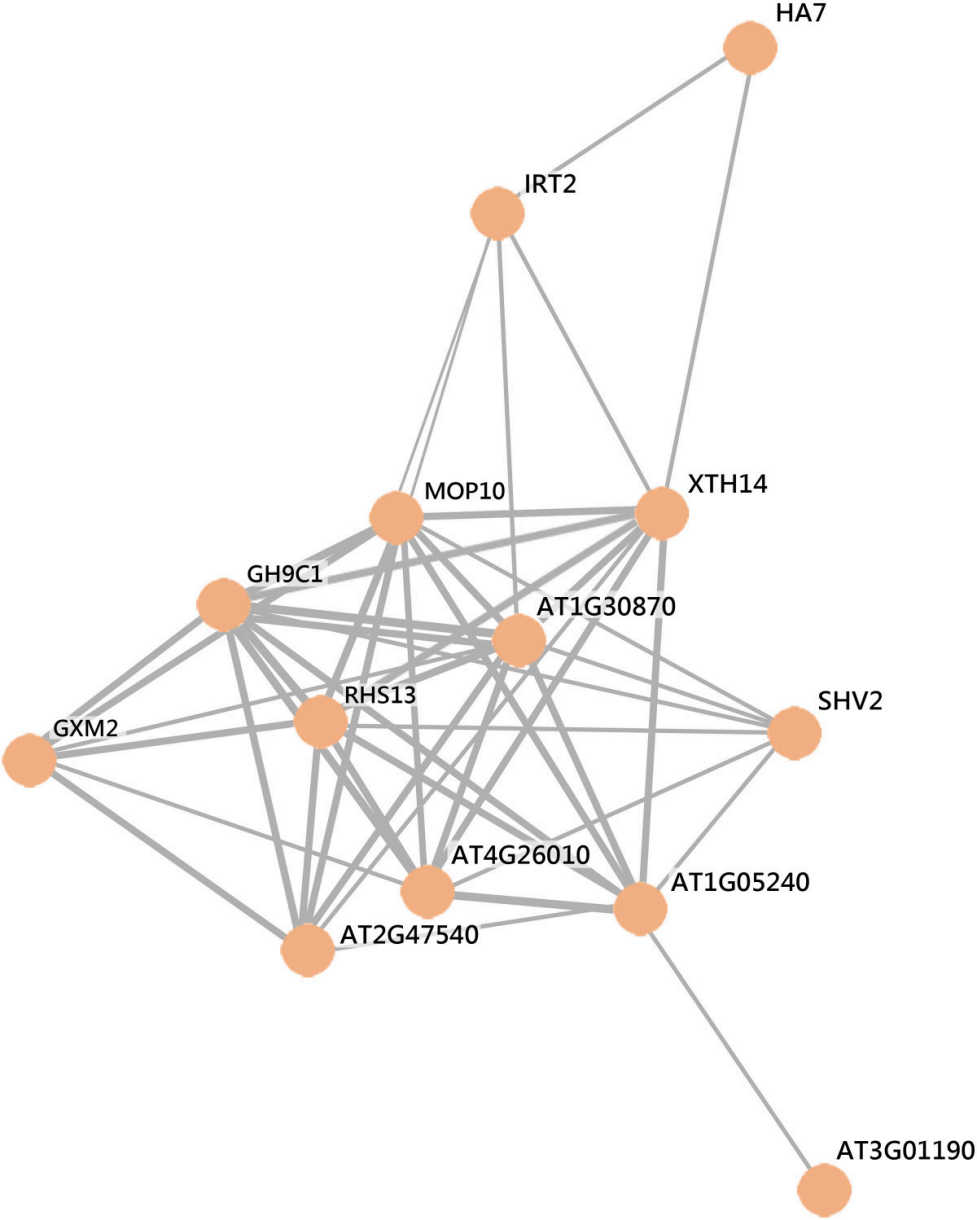
**Protein-protein interaction network of nutrient-responsive genes that are regulated at both transcript and protein levels**. The network was generated with STRING (http://string.embl.de) based on known and predicted interactions and displayed in confidence view.

Transcription is intrinsically stochastic and may not always reflect biologically significant changes in gene activity. Changes in both transcript and protein, on the other hand, can be regarded as being relatively little affected by noisy gene expression and can be used as a means to identify genes that are not much affected by noisy gene expression. For several transcriptionally regulated genes the cognate protein also showed changes in abundance in response to alteration in the nutrient regime. We define this subset as the “core trophomorphome.” To decipher functional modules of nutrient-responsive proteins *via* their interactions with each other, we constructed a protein-protein interaction (PPI) network of core trophomorphome (Figure 5). This network comprises the proline-rich protein-like PRPL1, the pollen Ole e 1 allergen and extensin family protein At2g47540, the glycosyl hydrolase 9C1 (GH9C1), the xyloglucan endotransglucosylase/hydrolase 14 (XTH14), the glucuronoxylan methyltransferase 2 (GXM2), the Fe^2+^ transporter IRT2, the proton ATPase HA7, the COBRA-LIKE protein 9 (COBL9), ROOT HAIR SPECIFIC 13 (RSH13), and the peroxidase superfamily proteins At3g01190, At1g05240, At1g30870, and At4g26010. GH9C1 is expressed in root hairs prior to bulge formation and during elongation, and was functionally associated with cell wall loosening during root hair morphogenesis (del Campillo et al., 2012). Similarly PRPL1, COBL9, and XTH14 are centrally involved in root hair elongation (Jones et al., 2006; Maris et al., 2009; Boron et al., 2014). The peroxidases At4g26010 (peroxidase 44) and At1g30870 (peroxidase 7) are annotated as being involved in cell wall organization and their transcripts are enriched in root hairs by 134- and 333-fold, respectively (Lan et al., 2013). Peroxidase 7 is auxin responsive and requires RHL4 for full expression (Yi et al., 2010; Bruex et al., 2012). Interestingly, peroxidase 7 is also responsive to Fe starvation at the protein but not at the transcript level, suggesting different modes of gene regulation in these two growth types. The two peroxidases are localized in the apoplast. RHS13 contains a pollen Ole e 1 allergen/extensin domain with high similarity to the root hair-specific proline-rich proteins PRP1/RHS7 and PRP3 and is localized in the apoplast. This gene carries the RHE (Root Hair Element) consensus sequence in its promoter (Kim et al., 2006; Won et al., 2009) and is highly specific for root hair cells (403-fold enrichment). Together with peroxidase 7, RHS13 belongs to the root hair-specific proteins, a group for which no corresponding peptides were detected in tissues other than root hairs (Lan et al., 2013).

**Figure 6:**
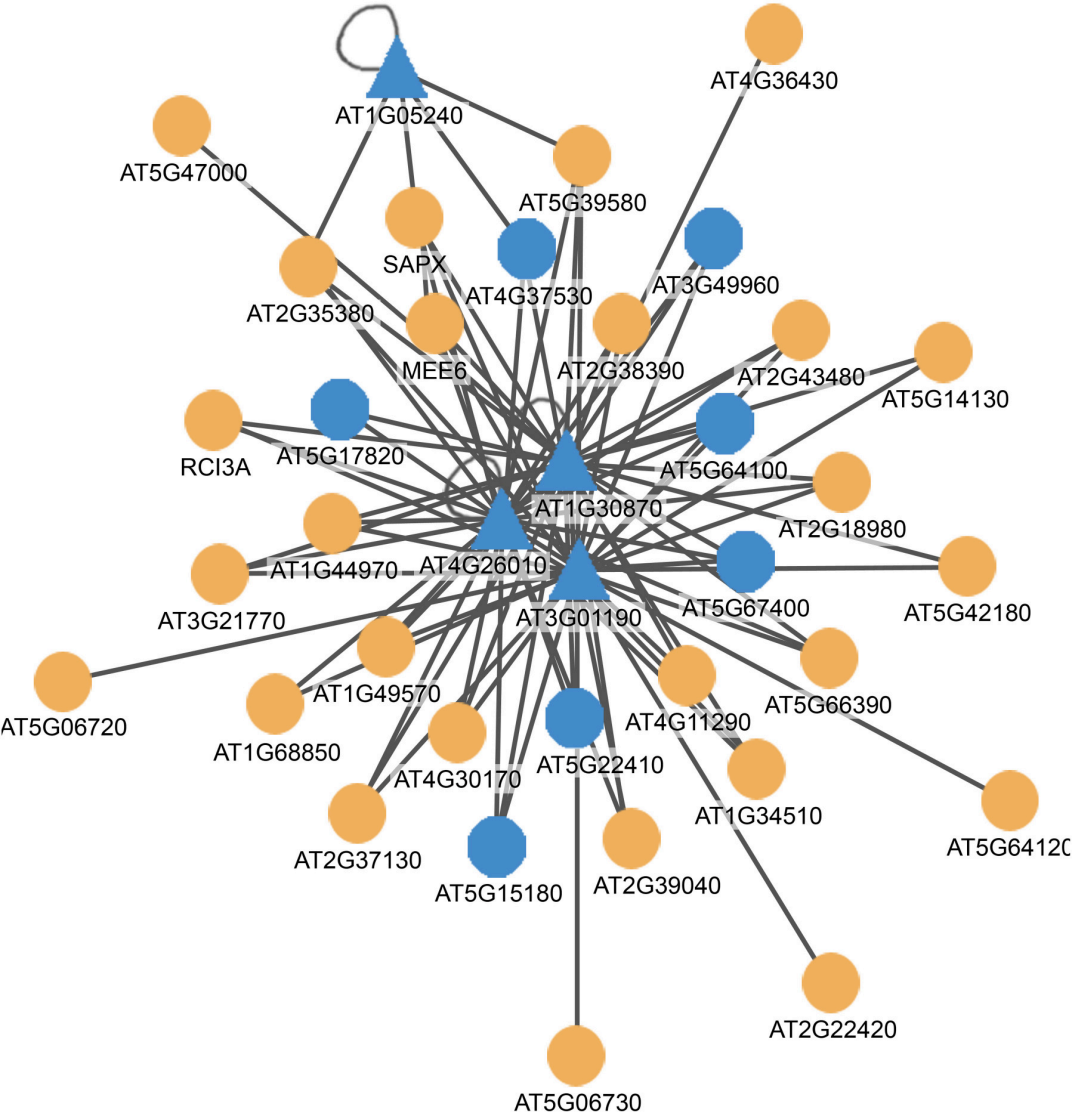
**Protein-protein interaction network illustrating putative interactions of query proteins (triangles) with bait proteins (circles)**. Blue nodes indicate proteins with root-hair-specific expression. The network was generated with the PAIR system (http://www.cls.zju.edu.cn/pair/).

To gain clearer insights into the function of the proteins in this cluster, we extended the PPI network by fishing putative interacting partners of these proteins. Using the proteins comprising the PPI network as an input to mine public PPI databases, 426 interactions were identified to the query proteins. One of the sub-clusters comprises 35 proteins based on the predicted interactions of four query proteins, the peroxidases At3g01190, At1g05240, At1g30870, and At4g26010 (Figure 6). Seven of the fished proteins are specifically expressed in root hairs, making interactions possible *in vivo*. Notably, all of the interacting proteins are peroxidases, indicating that ROS homeostasis is a central component of the trophomorphome. ROS distribution was shown to be critical for root hair development (Takeda et al., 2008; Sundaravelpandian et al., 2013). Pi deficiency changes the ROS concentration and distribution, and these changes were suggested to contribute to the morphological alterations induced by Pi starvation (Tyburski et al., 2009; Chacón-López et al., 2011). In particular, decreased H_2_O_2_ levels in response to Pi deficiency have been associated with meristem exhaustion, a hallmark response to Pi deficiency that attenuates longitudinal cell elongation, resulting in a shallower root system and an increase in root hair density per unit root length (Chacón-López et al., 2011; Savage et al., 2013). Notably, changes in ROS distribution appear to be nutrient specific (Shin et al., 2005), a prerequisite for the establishment of nutrient-specific phenotypes. It can be assumed that the PPI network shown in Figure 4 represents a set of core proteins that are critical in translating environmental information into alterations of the root hair phenotype.

## Conclusions

Mining public data sets that report root hair-specific proteomic or transcriptomic data and changes in the transcriptome/proteome upon nutrient starvation revealed different regulatory modes for transcripts and proteins enriched in root hair cells, which appear to be related to their function. This was an unexpected finding uncovering a cryptic regulatory layer that only becomes apparent when disparate omics levels are comparatively investigated. Considering root hair genes that are robustly changed in response to the nutritional regime, a key role for ROS metabolism and, as anticipated, cell wall-modifying proteins becomes obvious. Our study also sheds light on the apparent genetic redundancy of cell wall modification and other genes involved in root hair morphogenesis. Similar to what has been observed with the R3 MYB proteins CPC, ETC1, and TRY (Chen and Schmidt, 2015), some root hair-specific genes may be dispensable under control conditions but may be recruited for specific functions in response to environmental factors. For example, from the three highly similar xyloglucan endotransglucosylase/hydrolases XTH12, XTH13, and XTH14 only XTH12 and XTH14 are Pi-responsive, indicating more specific roles of the latter two proteins in inducing the root hair phenotype that is typical of Pi-deficient plants. On the other hand, most of the structural components of the cell wall such as proline-rich extension-like family proteins or leucine-rich repeat family proteins are root hair-specific but are not responsive to the nutrient regime.

The present inventory of nutrient-responsive root hair genes may soon be extended by data derived from proteomic or transcriptomic studies analyzing other growth-types that affect root hair morphogenesis. We believe that this catalog helps to identify important nodes in root hair trophomorphogenesis that have not been previously associated with this process.

## Materials and methods

### GO analysis and PPI network

Gene ontology (GO) enrichment analysis of genes sets was performed using the ClueGO version 2.0.7 plugin tool (Bindea et al., 2009) in Cytoscape version 3.2.1 (Shannon et al., 2003) with the GO Biological Process category. Overrepresented Biological Process categories were identified using an (right-sided) enrichment test based on the hypergeometric distribution. To correct the *P*-values for multiple testing Bonferroni step-down was used.

PPI networks of nutrient-responsive proteins were generated with STRING (http://string.embl.de) based on known and predicted interactions and displayed in confidence view. Extended PPI networks with nutrient-responsive query proteins were generated with the PAIR system (http://www.cls.zju.edu.cn/pair/).

## Author contributions

WS conceived the idea, analyzed data and drafted the manuscript. JS analyzed the data and revised the manuscript.

### Conflict of interest statement

The authors declare that the research was conducted in the absence of any commercial or financial relationships that could be construed as a potential conflict of interest.
